# Development of a Bicistronic Yellow Fever Live Attenuated Vaccine with Reduced Neurovirulence and Viscerotropism

**DOI:** 10.1128/spectrum.02246-22

**Published:** 2022-08-18

**Authors:** Hong-Jiang Wang, Yan Guo, Meng-Jiao He, Zhong-Yu Liu, Qing Ye, Xing-Yao Huang, Yong-Qiang Deng, Xiao-Feng Li, Cheng-Feng Qin

**Affiliations:** a Department of Research, The Chinese People’s Liberation Army Strategic Support Force Medical Center, Beijing, China; b Department of Virology, State Key Laboratory of Pathogen and Biosecurity, Beijing Institute of Microbiology and Epidemiology, Academy of Military Medical Sciences (AMMS), Beijing, China; c School of Medicine (Shenzhen), Sun Yat-Sen University, Guangzhou, China; d Guangzhou Eighth People’s Hospital, Guangzhou Medical University, Guangzhou, China; Shandong First Medical University

**Keywords:** yellow fever, IRES, bicistronic, live attenuated vaccines, neurovirulence, viscerotropism

## Abstract

The yellow fever (YF) live attenuated vaccine strain 17D (termed 17D) has been widely used for the prevention and control of YF disease. However, 17D retains significant neurovirulence and viscerotropism in mice, which is probably linked to the increased occurrences of serious adverse events following 17D vaccination. Thus, the development of an updated version of the YF vaccine with an improved safety profile is of high priority. Here, we generated a viable bicistronic YF virus (YFV) by incorporating the internal ribosome entry site (IRES) from *Encephalomyocarditis* virus into an infectious clone of YFV 17D. The resulting recombinant virus, 17D-IRES, exhibited similar replication efficiency to its parental virus (17D) in mammalian cell lines, while it was highly restricted in mosquito cells. Serial passage of 17D-IRES in BHK-21 cells showed good genetic stability. More importantly, in comparison with the parental 17D, 17D-IRES displayed significantly decreased mouse neurovirulence and viscerotropism in type I interferon (IFN)-signaling-deficient and immunocompetent mouse models. Interestingly, 17D-IRES showed enhanced sensitivity to type I IFN compared with 17D. Moreover, immunization with 17D-IRES provided solid protection for mice against a lethal challenge with YFV. These preclinical data support further development of 17D-IRES as an updated version for the approved YF vaccine. This IRES-based attenuation strategy could be also applied to the design of live attenuated vaccines against other mosquito-borne flaviviruses.

**IMPORTANCE** Yellow fever (YF) continually spreads and causes epidemics around the world, posing a great threat to human health. The YF live attenuated vaccine 17D is considered the most efficient vaccine available and helps to successfully control disease epidemics. However, side effects may occur after vaccination, such as viscerotropic disease (YEL-AVD) and neurotropic adverse disease (YEL-AND). Thus, there is an urgent need for a safer YF vaccine. Here, an IRES strategy was employed, and a bicistronic YFV was successfully developed (named 17D-IRES). 17D-IRES showed effective replication and genetic stability *in vitro* and high attenuation *in vivo*. Importantly, 17D-IRES induced humoral and cellular immune responses and conferred full protection against lethal YFV challenge. Our study provides data suggesting that 17D-IRES, with its prominent advantages, could be a vaccine candidate against YF. Moreover, this IRES-based bicistronic technology platform represents a promising strategy for developing other live attenuated vaccines against emerging viruses.

## INTRODUCTION

Yellow fever (YF) is a potentially fatal viral disease caused by infection with yellow fever virus (YFV). Infection with YFV leads to a variety of symptoms, ranging from subclinical to severe illness, including fever, nausea, vomiting, jaundice, hemorrhaging, and even death (1). YFV is transmitted by mosquitos and is mainly endemic to tropical regions, including Africa, Central America, and South America. It still represents a real threat to public health, leading to significant morbidity and mortality rates in human populations. Recent outbreaks emerged in Angola in 2015 to 2016 and Brazil in 2016 to 2019; in Brazil, there were more than 2,000 confirmed cases, including more than 500 deaths (2, 3).

There is no specific antiviral treatment against YFV infection; vaccination is the primary strategy for YF control. The live attenuated YF vaccine 17D (hereafter, 17D) is one of the most efficient live attenuated vaccines due to the very high rates of seroconversion and long-term protection following vaccination. Since its development in the 1930s, 17D has been administered to over 850 million people worldwide and has been successfully used to control the disease in many countries of endemicity (4). Although it is considered perfectly safe, vaccination with 17D causes mild adverse effects, such as myalgia, headache, and slight fever associated with low, transient viremia. In addition, serious adverse events (SAE), including YF vaccine-associated viscerotropic disease (YEL-AVD) and YF vaccine-associated neurologic disease (YEL-AND), have been reported during the past decades (5–10). YEL-AVD is a condition that mimics the initial symptoms of naturally acquired YF, including fever, headache, nausea, vomiting, and diarrhea. Patients with severe YEL-AVD may develop hypotension and hemorrhage, as well as acute renal and/or respiratory failure (11). YEL-AND manifests as several distinct clinical syndromes, including encephalomyelitis, meningoencephalitis, neuropathy, Guillain-Barre syndrome, or other symptoms of spinal myelitis (12). 17D retains a certain degree of neurotropism and neurovirulence for mice and nonhuman primates under laboratory conditions (13), which may contribute to the occurrence of YEL-AVD and YEL-AND. Thus, the development of an updated YF vaccine with reduced neurovirulence and viscerotropism is urgently needed.

YFV, together with other important human pathogens within the genus *Flavivirus* of the family *Flaviviridae*, including dengue (DENV), West Nile (WNV), Zika (ZIKV), and Japanese encephalitis (JEV) viruses. YFV has a 5′-m^7^GpppN-capped single-stranded positive-sense RNA genome approximately 11,000 nucleotides long. The viral genome encodes a single open reading frame (ORF) flanked by 5′ and 3′ untranslated regions (UTRs), which is translated into a polyprotein co- and posttranslationally cleaved by viral and host proteases to generate 10 mature viral proteins (C-prM-E-NS1-NS2A-NS2B-NS3-NS4A-NS4B-NS5). YFV is known to initiate its translation in a canonical cap-dependent manner. During this process, the eukaryotic initiation factor 4E (eIF4E), a component of the cap-binding complex eIF4F, recognizes an m^7^GpppN cap structure at the 5′ end of viral mRNAs (14). Only when bound to the cap structure can the eIF4F complex recruit the ribosome complex to the mRNA. Internal ribosome entry sites (IRESs) are defined as structural RNA elements that independently enable translation from a 5′ cap, which have been widely utilized to initiate the expression of an additional protein from a bicistronic vector. Among them, the *Encephalomyocarditis virus* (EMCV) IRES is functional in a variety of cultured cell types (15) and has a higher translation efficiency than other IRES sequences, including those from hepatitis A and C viruses (HAV and HCV), poliovirus (PV), human rhinovirus (HRV), and foot-and-mouth disease virus (FAMDV) (16). The EMCV IRES is commonly used in current bicistronic vectors (17). Due to the difference in the efficiency of translation initiation between EMCV IRES-dependent and cap-dependent gene expression, an imbalance of protein expression was achieved in the bicistronic virus, resulting in virulence attenuation without any change in viral proteins (18–20). Thus, the strategy of IRES insertion in the virus has been widely employed in viral vaccine designs, such as those for Chikungunya virus (CHIKV), tick-borne encephalitis virus (TBEV), and Venezuelan equine encephalitis virus (VEEV) (21–23).

Here, we rationally designed an updated YF vaccine by incorporating the EMCV IRES into the infectious clone of 17D. Compared to the licensed 17D vaccine, the resulting bicistronic recombinant 17D-IRES has an attenuated neurovirulence and viscerotropism phenotype, with induction of a solid protective immune response against lethal virus challenges similar to the 17D vaccine. Our findings support further development of 17D-IRES as a next-generation YF vaccine in a nonhuman primate model and clinical trials.

## RESULTS

### Construction and *in vitro* characterization of the recombinant 17D-IRES virus.

**FIG 1:**
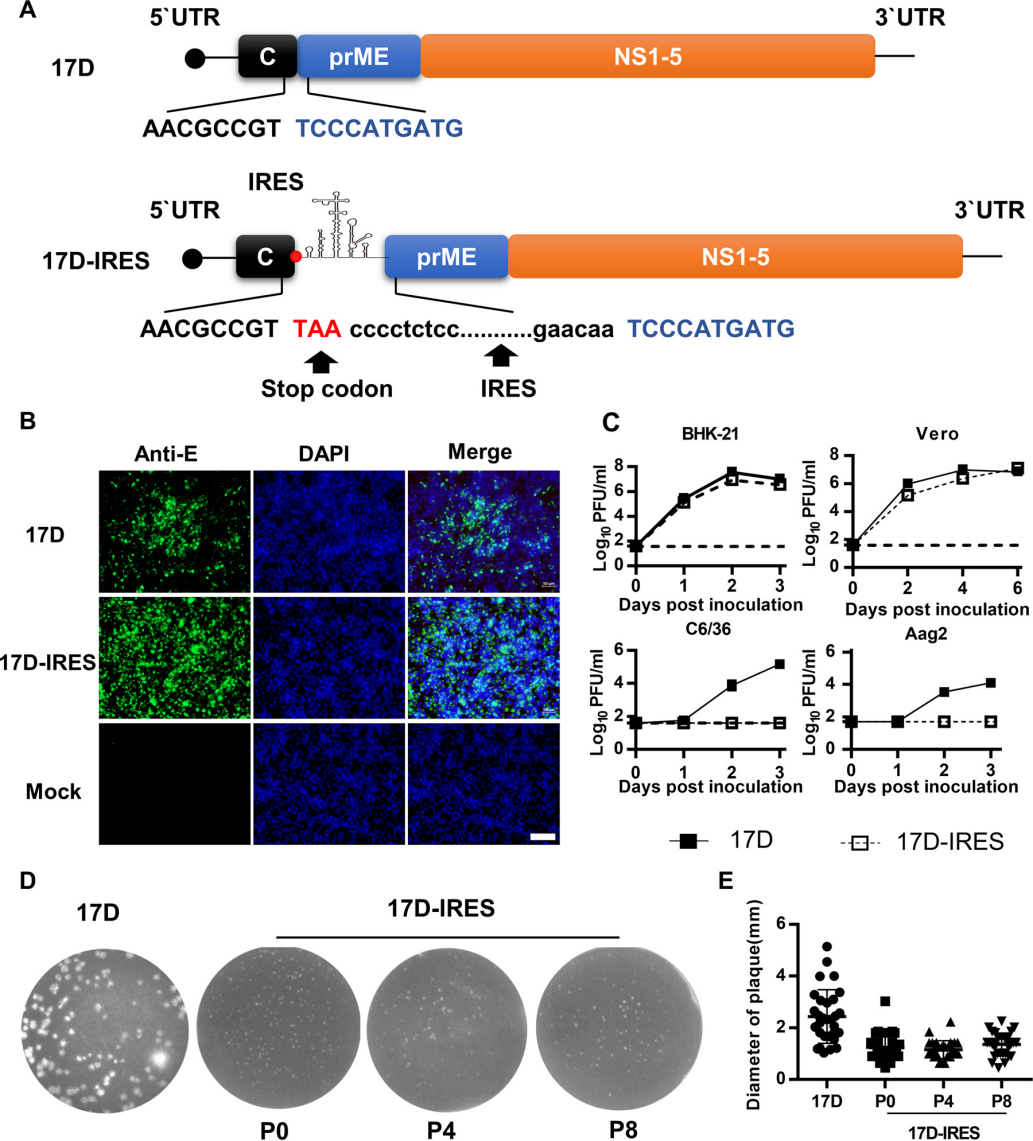
Construction and characterization of 17D-IRES. (A) Graphic representation of the genomic constructs of 17D and 17D-IRES. The EMCV IRES (black dots) was inserted into the 17D genome. The termination codon TAA (red, uppercase) followed by EMCV IRES was inserted downstream of the C protein. (B) BHK-21 cells were infected with the indicated viruses at an MOI of 0.01; E protein expression of viruses was detected by IFA with the flavivirus-specific monoclonal antibody (MAb) 2A10 at 36 h postinfection. Bar, 100 μm. (C) Growth curves of 17D and 17D-IRES in BHK-21, Vero, C6/36, and Aag2 cells. Cells were infected with the indicated viruses at an MOI of 0.01. The cell supernatants were collected at the indicated times for the determination of virus titers by plaque assay using BHK-21 cells. The growth curve data represent the means and standard deviation (SD) of three independent experiments. (D) Plaque morphologies of 17D or 17D-IRES from different passages (P0, i.e., before passaging; P4; and P8) on BHK-21 cells on day 3 postinoculation. (E) Viral plaque diameters were measured with a standard ruler in centimeters (30 plaques/virus).

To generate a recombinant bicistronic 17D containing the EMCV IRES (named 17D-IRES), the termination codon (TAA) followed by EMCV IRES was inserted downstream of the C protein based on the infectious clone of 17D (Fig. 1A). This strategy allowed translation of the C protein to occur by a 5′ cap-dependent mechanism, while translation of all the other viral proteins is mediated by the IRES. As expected, recombinant 17D-IRES was successfully recovered in BHK-21 cells. Indirect immunofluorescence assay (IFA) results showed that 17D-IRES exhibited a similar viral protein expression pattern in BHK-21 cells to that of 17D (Fig. 1B). Growth curves showed that 17D-IRES replicated as efficiently as 17D in BHK-21 and Vero cells (Fig. 1C). In contrast, 17D-IRES replication was highly restricted in C6/36 and Aag2 cells, as demonstrated by the detection of no viable virus during the observation time (Fig. 1C). To test its genetic stability, the 17D-IRES virus was serially passaged 8 times in BHK-21 cells. As shown in Fig. 1D and E, 17D-IRES retained a homogeneous small-plaque phenotype. Sanger sequencing confirmed that no nucleotide substitutions were identified in the whole genome of 17D-IRES. The results above demonstrate that 17D-IRES is genetically stable in mammalian cell lines but not viable in mosquito cells.

### 17D-IRES showed significantly reduced neurovirulence in comparison with 17D.

**FIG 2:**
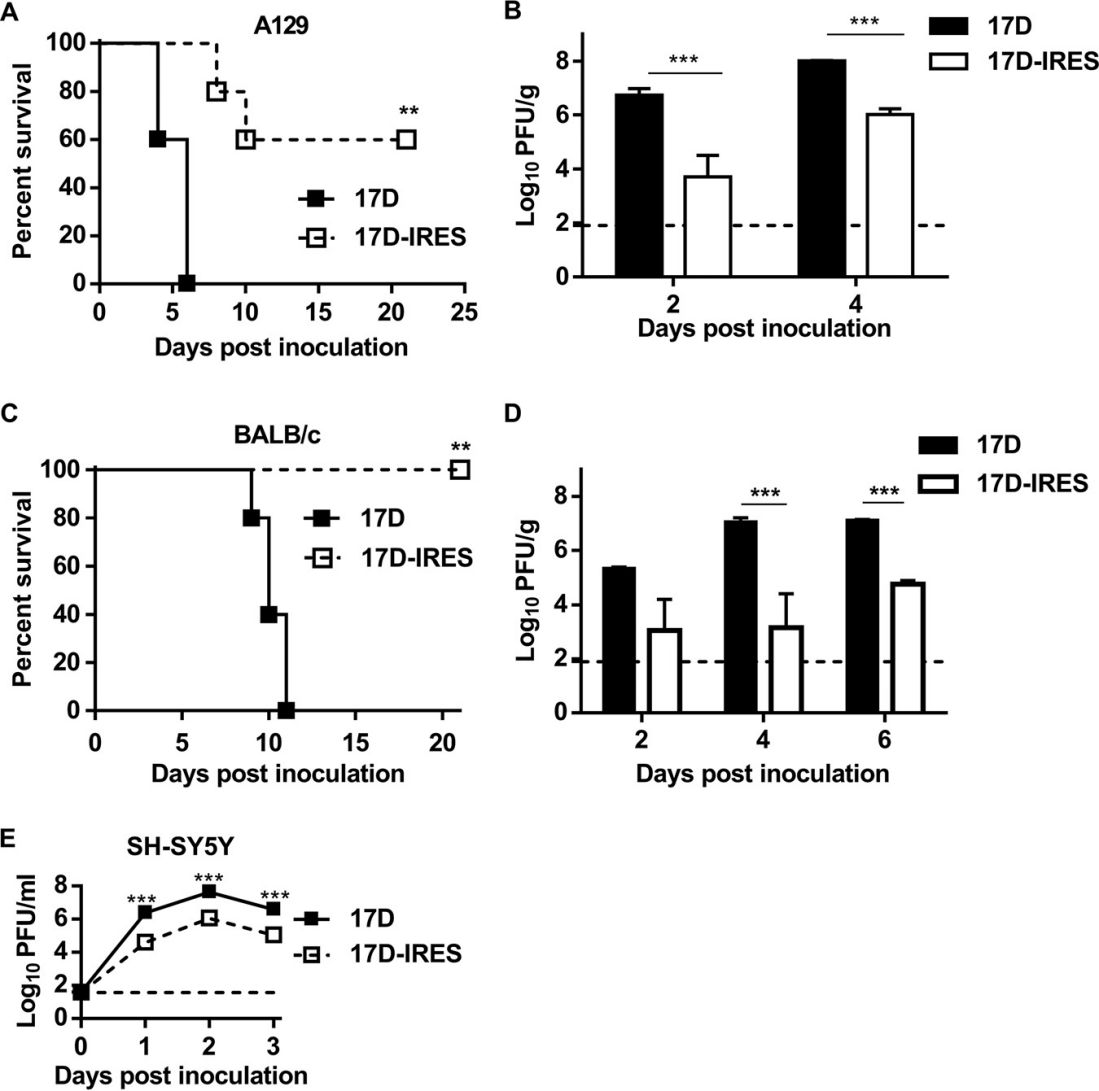
17D-IRES is highly attenuated in neurovirulence in BALB/c and A129 mice. Four-week-old A129 mice (A and B) and 3-week-old BALB/c mice (C and D) were i.c. inoculated with 10^3^ PFU of 17D or 17D-IRES. Survival curves of the animals were observed (A and C), and viral loads in mouse brains were determined by plaque assay (B and D). The limit of detection is shown as a dashed line with a value of 89 PFU/g. Data on the viral titer in the brain represent the means and SD of three independent experiments and were measured via unpaired *t* test. For survival analysis, Kaplan-Meier survival curves were analyzed by a log-rank test. (E) Growth curves of 17D and 17D-IRES in SH-SY5Y cells. Cells were infected with the indicated viruses at an MOI of 0.01. The cell supernatants were collected at the indicated times for determination of the virus titers by plaque assay using BHK-21 cells. Growth curve data represent the means and SD of three independent experiments. *, *P* < 0.05; **, *P* < 0.01; ***, *P* < 0.001.

A129 mice have been well established for neurovirulence tests (24). We first compared the neurovirulence of 17D-IRES and 17D in an A129 mouse model. As expected, intracranial (i.c.) injection with 10^3^ PFU of 17D resulted in 100% lethality, with an average survival time (AST) of 5.3 days, supporting that 17D retained significant neurovirulence in mice (Fig. 2A). In contrast, 60% (3 of 5) of the mice that received the 17D-IRES challenge survived, with an AST of 9.0 days (Fig. 2A). More importantly, infectious virus titers in I7D-IRES-inoculated mouse brains were significantly lower than those in 17D-inoculated animals, as demonstrated by over 1,000-fold reduction at the indicated time points (Fig. 2B). We further compared the neurovirulence of 17D-IRES and 17D in immunocompetent BALB/c mice. As expected, i.c. inoculation with 10^3^ PFU of 17D resulted in 100% mortality in the BALB/c mice with typical neurological symptoms, while all mice inoculated with 17D-IRES survived, and no clinical symptoms were observed (Fig. 2C). Importantly, the viral titers in the 17D-IRES-infected mouse brains were significantly lower than those of the 17D-infected ones, with a 100- to 10,000-fold reduction during the observation period (Fig. 2D). All these data demonstrate that the 17D-IRES virus displayed significantly decreased neurovirulence in mice in comparison with 17D. We further tested the replication of 17D-IRES and 17D in neuronal SH-SY5Y cell lines. As shown in Fig. 2E, 17D-IRES displayed significantly reduced replication compared to 17D, indicating that insertion of the IRES element resulted in attenuation of replication of 17D in neuronal cells.

### 17D-IRES displayed limited viscerotropism in AG6 mice.

**FIG 3:**
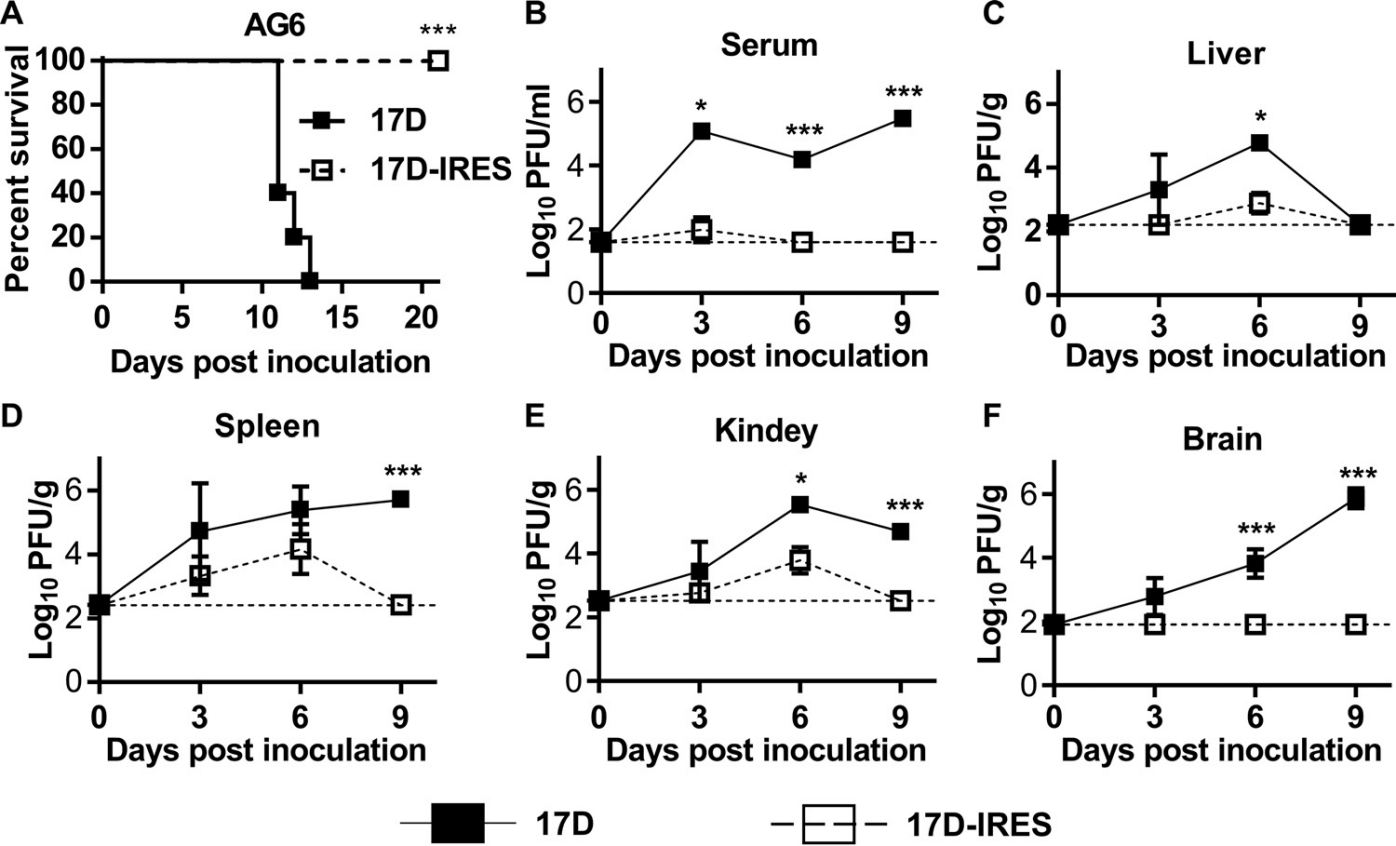
17D-IRES is highly attenuated in viscerotropism in AG6 mice. (A) Survival curve of AG6 mice infected with 17D or 17D-IRES; 6-week-old AG6 mice were infected with 10^4^ PFU of 17D or 17D-IRES by the i.p. route. Kaplan-Meier survival curves were analyzed by a log-rank test. (B) Replication of 17D and 17D-IRES in mouse serum. (C to F) Replication of 17D and 17D-IRES in mouse tissues. The virus loads were tittered at the indicated times by a standard plaque-forming assay performed on BHK-21 cells. Data represent the means and SD of three independent experiments. Data on the viral titer were measured via unpaired *t* test. *, *P* < 0.05; **, *P* < 0.01; ***, *P* < 0.001.

Mice deficient in alpha/beta and gamma interferon (IFN-α/β and -γ) receptors have been widely used for viscerotropism testing (25). To assess the viscerotropism of 17D-IRES, groups of AG6 mice were inoculated with 10^4^ PFU of 17D-IRES or 17D through the intraperitoneal (i.p.) route. As shown in Fig. 3A, the 17D-inoculated mice appeared thin and depressed and died within 13 days, with an AST of 11.6 days. In contrast, all the 17D-IRES-inoculated mice survived with no clinical symptoms during the observation period. High levels of infectious virus in serum were detected in 17D-inoculated mice during the observation period, whereas transient viremia was measurable on day 3 postinoculation with 17D-IRES, which dropped to below the limit of detection (LOD) on days 6 and 9 postinoculation (Fig. 3B). Similarly, viral loads in the spleen, liver, and kidneys were significantly higher in the 17D-inoculated mice than those of the 17D-IRES-inoculated mice (Fig. 3C to F). Notably, 17D replicated efficiently in the mouse brain and peaked at 10^6^ PFU/g on day 9 postinoculation, when the mice began to die (Fig. 3F). In contrast, no measurable infectious viruses were observed in the brains of the 17D-IRES-inoculated mice (Fig. 3F). Together, these results demonstrate that the 17D-IRES virus is highly attenuated in virulence with a significantly weaker viscerotropic phenotype than that of the 17D vaccine in AG6 mice.

### 17D-IRES showed increased sensitivity to the IFN.

**FIG 4:**
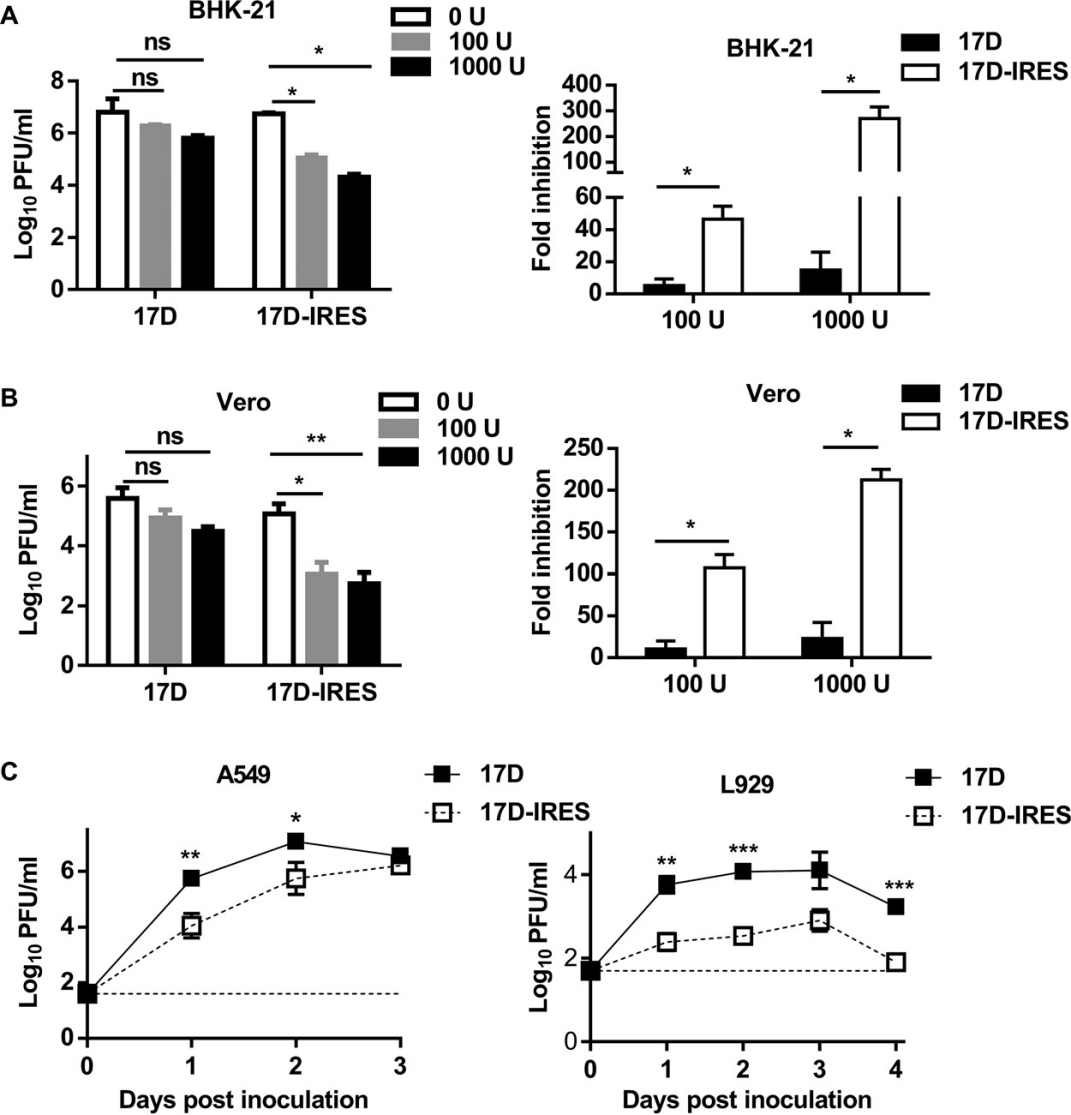
17D-IRES shows increased sensitivity to IFN. An IFN sensitivity assay was performed on BHK-21 (A) and Vero cells (B). Cells were treated with media containing different concentrations of IFN-αA/D (0, 100, or 1,000 U/mL). The treated cells were washed with PBS and infected with 17D or 17D-IRES at an MOI of 1. At 24 h postinfection, the supernatants were collected, and the virus titers were determined by plaque assay in BHK-21 cells. The fold changes of the virus titers were also calculated (right). (C) Growth curves of 17D and 17D-IRES on A549 (left) and L929 (right) cells. Monolayers of A549 and L929 cells were infected with the indicated viruses at an MOI of 0.01. The cell supernatants were collected at the indicated times for the determination of the virus titers by plaque assay using BHK-21 cells. Data represent the means and SD of three independent experiments. Viral titer data were measured via unpaired *t* test. n.s., not significant; *, *P* < 0.05; **, *P* < 0.01.

The IRES attenuation strategy was presumed to imbalance translation of the viral protein (26). In our study, 17D-IRES presented attenuated neurovirulence in BALB/c mice compared with A129 mice. We hypothesized that the highly attenuated neurovirulence of 17D-IRES was partly due to its sensitivity to the IFN-I system, which exerts a neuroprotective function against viral infections. To test this hypothesis, the growth curves of 17D and 17D-IRES were measured under gradient doses of IFN-I treatment in BHK-21 and Vero cells, which are recognized to be deficient in IFN production but retain an intact response to IFN. As shown in Fig. 4A, the 17D infectious viral particle levels remained relatively stable over 24 h of treatment and were comparable to levels in untreated cells. However, 17D-IRES was more sensitive to inhibition of viral replication by IFN, with reduced viral production in a dose-dependent manner. In contrast, a similar dose-dependent IFN sensitivity was observed in Vero cells (Fig. 4B). To further assess the sensitivity of 17D-IRES to the IFN-I system, the replication of 17D-IRES and 17D was tested in immunocompetent A549 human epithelial cells and murine fibrosarcoma L929 cells, both of which have a functional IFN-α/β receptor. As expected, replication of the 17D-IRES virus was significantly attenuated in comparison with the 17D virus (Fig. 4C). These data demonstrate that 17D-IRES displays a higher sensitivity to the antiviral actions of IFN than does 17D.

### 17D-IRES elicited protective immune responses against YFV.

**FIG 5:**
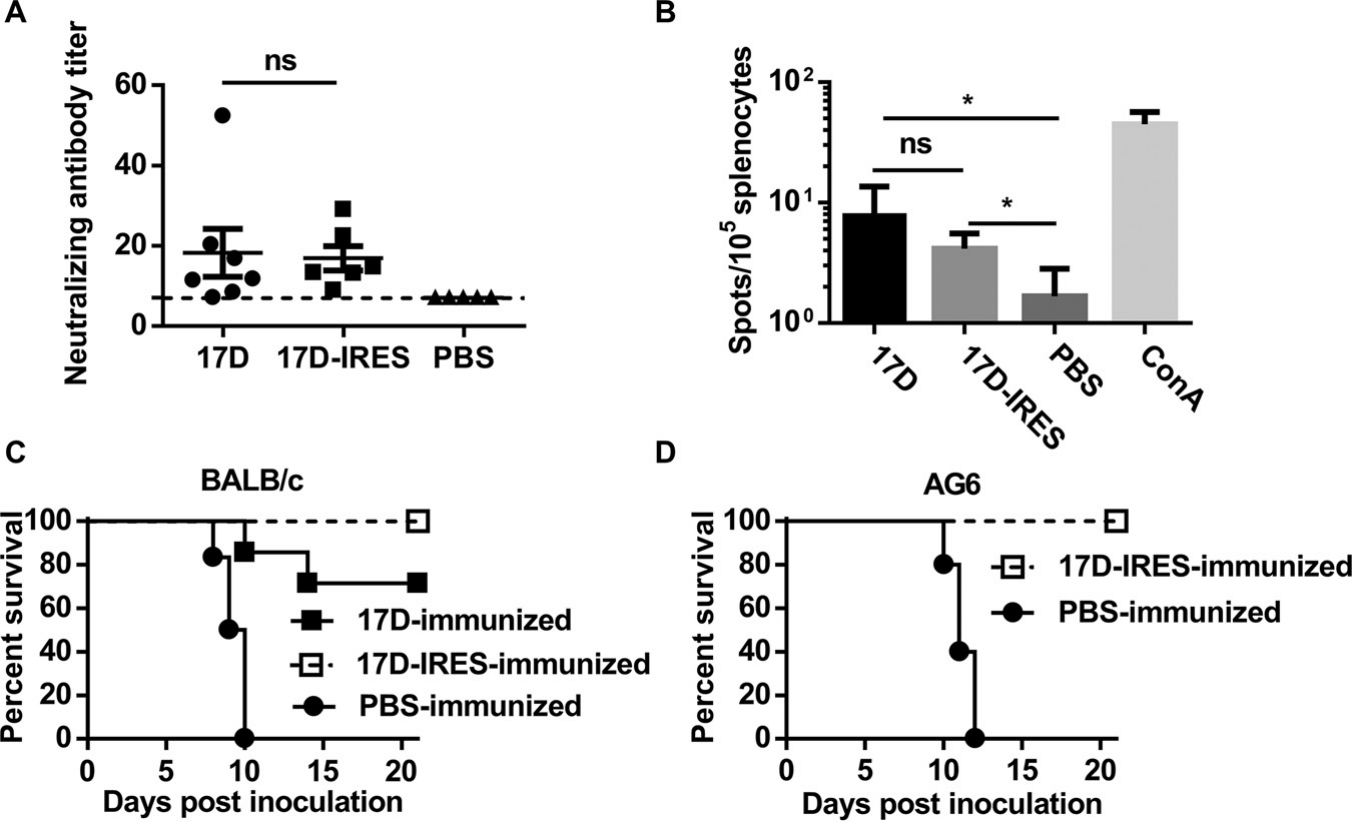
17D-IRES elicits YFV-specific immune responses and provides full protection in mice. (A) Neutralizing antibody responses in mice inoculated with 17D or 17D-IRES. Sera were collected from the immunized mice after 28 days postimmunization to detect neutralizing antibody titers against 17D by PRNT_50_. The dotted line represents the limits of detection of PRNT_50_. Significance was calculated using two-way analysis of variance (ANOVA) with multiple-comparison tests. (B) Detection of protective T-cell responses directed against 17D. ELISpot assay data showing IFN-γ production by splenocytes of BALB/c mice vaccinated with 10^5^ PFU of 17D or 17D-IRES, following 36 h of *ex vivo* restimulation with the lysate of 17D-infected BHK-21 cells. Significance was calculated using unpaired *t* test. (C) Survival curve of the 17D-IRES-immunized BALB/c mice challenged with 17D through the i.c. route. (D) Survival curve of 17D-IRES-immunized AG6 mice challenged with 17D through the i.p. route. Kaplan-Meier survival curves were analyzed by a log-rank test. n.s., not significant; *, *P* < 0.05; **, *P* < 0.01; ***, *P* < 0.001.

Next, we investigated the immunogenicity of 17D-IRES. As shown in Fig. 5A, subcutaneous (s.c.) immunization of adult BALB/c mice with 17D-IRES induced high levels of neutralizing antibodies (geometric mean titer [GMT], 17.0) on day 28 postimmunization, consistent with immunization with 17D (GMT, 18.3). To detect T-cell immune responses induced by the vaccination, a murine IFN-γ enzyme-linked immunospot (ELISpot) assay was conducted. The results showed that comparative numbers of IFN-γ-releasing cells were detected in both 17D-IRES- and 17D-immunized mice (Fig. 5B), indicating that 17D-IRES vaccination is capable of inducing potent cell-mediated immune responses against YFV antigens, similarly to the 17D vaccine.

Finally, we examined the protection efficiency of the highly attenuated 17D-IRES virus against virus challenges. The lethal challenge study showed that all the 17D-IRES-immunized BALB/c and AG6 mice survived without any clinical symptoms during the observation period (Fig. 5C and D), demonstrating that 17D-IRES confers full protection in mice against lethal YFV challenge.

These data show that 17D-IRES is capable of effectively inducing humoral and cellular immunity, providing full protection against lethal YFV challenge.

## DISCUSSION

The development of new attenuated vaccines is an important aspect of YFV research. Here, we report 17D-IRES, a recombinant 17D vaccine carrying the IRES of EMCV, which displays a highly attenuated neurovirulence in mice. The 17D-IRES virus is highly sensitive to IFN-I treatment and exhibited much less viscerotropism than the 17D vaccine in AG6 mice. More importantly, immunization with the highly attenuated 17D-IRES can still provide complete protection against the virus challenge.

The main goal of our study was to attenuate the neurovirulence and potent viscerotropism of the 17D vaccine but to maintain its immunogenicity. Using reverse genetics, we inserted the EMCV IRES into the 17D vaccine genome to make initiation of translation of the viral C protein and all other viral proteins gene 5′ cap and IRES dependent, respectively. This IRES-based rational attenuation strategy targets the level and balance of protein production and has been developed for the alphaviruses chikungunya virus and Venezuelan equine encephalitis virus and the flavivirus Murray Valley encephalitis virus (21, 27, 28). For flaviviruses with monocistronic genome organization, the insertion of IRES resulted in an imbalance in the relative amounts of virus proteins from a single precursor, which was thought to partly contribute to the attenuated phenotypes of viruses (26). As expected, the average plaque size of 17D-IRES was significantly smaller than the plaque size of the parental viruses (Fig. 1D and E). The small-plaque phenotype of 17D-IRES indicates its potential attenuation in comparison with its parental 17D viruses. In general, the reduction of the plaque phenotype is positively correlated with the attenuation of the virus, which was confirmed in several studies (29, 30). In particular, the bicistronic 17D-IRES exhibited the uncoupling of the coordinated cleavages at the C-prM junction and displayed significantly decreased replication in suckling mouse brains compared to 17D. In addition, host IFN signaling is the major barrier to the viscerotropism of neurotropic flaviviruses. For example, there exists evidence supporting a protective function of IFN-I against viral infections in the central nervous system (CNS), including ZIKV and herpes simplex virus (31, 32). Indeed, our data also showed that the IRES insertion rendered 17D-IRES more sensitive to IFN-β pretreatment in comparison with 17D, which partly accounts for the attenuated neurovirulence of 17D-IRES in mice. Moreover, even in immunodeficient mice defective in innate immune responses, the 17D-IRES virus showed high attenuation in viscerotropism compared to the 17D vaccine. Notably, viremia after 17D-IRES vaccination was transiently present at a very low level in immunocompromised AG6 mice. Additionally, 17D-IRES was unable to replicate in mosquito cells but replicated efficiently in Vero cells, with high genetic stability in BHK-21 cells. These findings support the large-scale production of 17D-IRES in the future. To our surprise, despite its increased attenuation, the 17D-IRES virus still exhibited efficient induction of humoral immunity. Using a two-dose vaccination regimen, comparable neutralizing antibody responses of a magnitude exceeding that considered protective (50% plaque reduction/neutralization titer [PRNT_50_], >10) were developed in all mice immunized with 17D or 17D-IRES. More importantly, 17D-IRES immunization provided complete protection against i.c. challenge with 17D in mice, while 17D conferred partial protection against challenge with the same dose of 17D.

In summary, we generated a recombinant replication-competent 17D-IRES that carries IRES and that is notably less neurovirulent, neurotropic, and viscerotropic than the 17D vaccine. To our knowledge, this is the first report on attenuation of the 17D vaccine using the IRES-based attenuation strategy. This promising vaccine candidate is worth being tested in nonhuman primates to determine if it is suitable for evaluation in humans.

## MATERIALS AND METHODS

### Cells and viruses.

BHK-21 cells (ATCC CCL-10), L929 cells (ATCC CCL-1), SH-SY5Y cells (ATCC CRL-2266), and Vero cells (ATCC CCL-81) were cultured in Dulbecco’s minimal essential medium (DMEM; Invitrogen). A549 cells (ATCC CCL-185) and Aedes albopictus C6/36 cells (ATCC CRL-1660) were cultured in RPMI 1640. Aedes aegypti cell line Aag2 (ATCC CCL-125) was cultured in Schneider’s insect medium (Gibco). All the media above were supplemented with 10% fetal bovine serum (FBS; Gibco), 100 U/mL penicillin, and 100 μg/mL streptomycin. BHK-21, Vero, L929, and A549 cells were grown at 37°C in 5% CO_2_, whereas the C6/36 and Aag2 cell lines were grown at 28°C. The live attenuated vaccine 17D (GenBank accession no. MN072725) was recovered from the infectious plasmid pYF-17D (33). The viral stock was prepared and titrated using the standard plaque-forming assay in BHK-21 cells and stored at –80°C for further use.

### Plasmid construction.

The plasmid was constructed using the standard molecular biology protocols based on pYF-17D. To construct the IRES-based 17D infectious clone (named p17D-IRES), the EMCV IRES was amplified by PCR using plasmid pIRES-neo as a template. The EMCV IRES was inserted into the 17D genome just between the capsid and prM. The stop cordon (ATT) was added at the 3′ ends of the capsid gene to terminate the translation. The primers used here are shown in Table S1 in the supplemental material.

### Transcription and transfection.

Plasmid p17D-IRES (10 μg) was linearized with *XhoI* (NEB), purified with phenol-chloroform, ethanol precipitated, and dissolved in RNase-free water. The linearized DNA was used as a template for *in vitro* transcription using the RiboMAX large-scale RNA production system (Promega) in the presence of the cap analog m^7^GpppA (Promega). RNA transcripts (5 μg) were purified using a PureLink RNA minikit (Thermo Fisher Scientific) and transfected into BHK-21 cells seeded into 6-well plates using Lipofectamine 3000 reagent (Invitrogen). Supernatants were then collected on day 4 posttransfection when a typical cytopathic effect (CPE) was observed.

### Indirect immunofluorescence assay.

IFAs were performed as the standard protocols. Briefly, cells were previously seeded into a 24-well plate and infected with the virus at a multiplicity of infection (MOI) of 0.01. At 36 h postinfection, the culture supernatants were removed, and the cells were fixed with ice-cold acetone and incubated with monoclonal antibody (2A10) against flavivirus E protein at 37°C for 1 h (34). After washing three times in phosphate-buffered saline (PBS), cells were incubated with the second antibody conjugated with Alexa fluor 488 (ZSGB-BIO) for 45 min. The nucleus was stained with 4′,6-diamidino-2-phenylindole (DAPI; Invitrogen), and the positive cells were examined under a fluorescence microscope (Zeiss).

### Growth curves.

Growth curves of 17D or 17D-IRES were measured on indefinite cells. The cells were seeded into the 24-well plate and infected with 17D or 17D-IRES at an MOI of 0.01. Culture supernatants were collected at successive 1-day intervals. Progeny viral titers were tittered by plaque assay on BHK-21 cells.

### Genetic stability assay.

17D-IRES was serially passaged in BHK-21 cells at an initial MOI of 0.01. Viral RNA was extracted from the fourth and eighth passages and sequenced. The plaque phenotypes were evaluated by plaque assay in BHK-21 cells.

### IFN sensitivity assay.

BHK-21 cells or Vero cells were previously seeded into the 96-well plate. The monolayer cells were incubated with a medium containing various doses of IFN-αA/D (0, 100, or 1,000 U/mL; Gibco) for 6 h. The supernatants were removed, and the cells were washed with PBS, followed by incubation with 17D or 17D-IRES at an MOI of 1 for 1 h at 37°C. Then, the unbound virus was removed by gentle washing with PBS. After 24 h of incubation, the supernatants were collected, and virus titers were determined by plaque assay in BHK-21 cells.

### Neutralization assay.

17D-specific neutralizing antibodies were measured using a standard 50% plaque reduction/neutralization test (PRNT_50_) (35). Briefly, serum was heat-inactivated for 30 min at 56°C. A 1:8 serum dilution was prepared in DMEM containing 2% FBS, and serial 2-fold dilutions were mixed with equal volumes of viral solution to yield a mixture containing ~500 PFU of virus per mL. After incubation at 37°C for 1 h, 250-μL volumes of the virus-antibody mixtures were added to the wells of 12-well plates containing confluent monolayers of BHK-21 cells. Viral titers were then determined using the plaque assay described above. The endpoint neutralization titer was calculated using the Spearman-Karber method (36).

### Enzyme-linked immunosorbent spot assay.

ELISpot was performed using a mouse IFN-γ ELISpot assay (Mabtech) under the manual standard. In brief, mice were sacrificed, and single-cell suspensions of splenocytes were obtained. Sham-vaccinated and vaccinated mouse splenocytes (10^5^ per well) were each incubated with 10^5^ PFU of heat-inactivated 17D that was prepared in BHK cells at 37°C for 24 h, and IFN-γ spots were developed and counted (AT-Spot ELISpot analysis system). Data were normalized by subtracting the number of spots from samples incubated with noninfected BHK cell lysates.

### Ethics statement.

All animal experiments were approved by and conducted in strict accordance with the guidelines of the Institutional Experimental Animal Welfare and Ethics Committee (IACUC-13-2016-001). The BALB/c mice used in this study were purchased from Beijing Vitalriver Laboratory Animal Technology Co., Ltd. 129/Sv/Ev mice deficient in IFN-α/β receptors (A129 mice) and C57BL/6 mouse deficient in types I and II interferon (IFN) receptors (AG6 mice) were maintained at the Laboratory Animal Center, AMMS.

### Mouse experiments.

To examine the attenuation of 17D-IRES in neurovirulence, groups of 4-week-old A129 mice or 3-week-old BALB/c mice were inoculated with 10^3^ PFU of 17D or 17D-IRES through the intracranial (i.c.) route and monitored for clinical symptoms and mortality for 21 days. Mouse brains were collected for viral detection on days 2, 4, and 6 postinfection.

To test the attenuation of 17D-IRES in viscerotropism, groups of 6-week-old AG6 mice were intraperitoneally (i.p.) inoculated with 10^4^ PFU of 17D or 17D-IRES and monitored for 21 days. Mouse tissues were collected for viral detection on days 3, 6, and 9 postinfection. The virus titer in each supernatant was determined by plaque assay on BHK-21 cells.

To assess the immunogenicity of 17D-IRES, groups of 4-week-old BALB/c mice were immunized with 10^5^ PFU of 17D or 17D-IRES through the s.c. route and boosted 1 month later. Mice immunized with PBS were used as the control. All the mice were bled by tail vein puncture 4 weeks after the boost immunization. The sera were stored at –20°C for determination of neutralizing antibodies by PRNT_50_. To assess the cellular immunity induced by 17D-IRES, groups of 4-week-old BALB/c mice were immunized with 10^5^ PFU of 17D or 17D-IRES through the s.c. route. On day 28 postimmunization, the mice were executed and spleens were collected for cellular immunity assay by ELISpot.

Intracranial (i.c.) challenge has been used previously to evaluate the protection conferred by YF17D and YF17D-derived vaccines in BALB/c mice (37). For the protection assay in BALB/c mice, groups of 4-week-old BALB/c mice were immunized with 10^5^ PFU of 17D through the s.c. route and boosted 1 month later. On day 28 postimmunization, the mice were challenged with 100 PFU of 17D by the i.c. route and monitored for clinical symptoms and mortality for 21 days.

For the protection assay in AG6 mice, groups of 4-week-old AG6 mice were intraperitoneally (i.p.) inoculated with 10^4^ PFU of 17D-IRES. On day 28 postimmunization, the immunized AG6 mice were challenged by the i.p. route with 10^4^ PFU of 17D; all mice were observed for clinical signs and death for 21 days.

### Statistical analysis.

All data were analyzed using standard GraphPad Prism version 8.0 software. Quantitative data were represented as means ± standard deviations and obtained from at least three independent experiments. For *in vitro* experiments, an unpaired *t* test was performed for comparison between 2 groups, and two-way ANOVA was conducted with multiple-comparison tests for multiple comparisons between groups. For survival analysis, Kaplan-Meier survival curves were analyzed by a log-rank test. Values were considered statistically significantly different at *P* < 0.05, *P* < 0.01, and *P* < 0.001.
