# Supplementary material for: Development of a Bicistronic Yellow Fever Live Attenuated Vaccine with Reduced Neurovirulence and Viscerotropism
Source: Microbiol Spectr. 2022 Aug 18;10(5):e02246-22. doi: 10.1128/spectrum.02246-22 (PMC9602263; doi:10.1128/spectrum.02246-22)
Supplement: Supplemental file 1 — Supplemental material. Download spectrum.02246-22-s0001.pdf, PDF file, 0.01 MB [file spectrum.02246-22-s0001.pdf]

Table S1. Primers for construction of 17D-IRES used in this study.

Primer	Sequence (5`-3`)
NOT1-F	CGACGCGGCCGCGCTAGCGATGAC
17D-IRES-R1	GGGAGAGGGGTTAACGGCGTTTCCTTGAGGACAATC
IRES-F	GGAAACGCCGTTAACCCCTCTCCCTCCCCCCCCCCT
IRES-R	GGGATTGTTCCATGGTTGTGGCCATATTATC
17D-IRES-F2	GGGATTGTTCCATGGTTGTGGCCATATTATC
Nsi1(-)	GGCCACAACCATGGAACAATCCCATGATGTTC TGACTGTGCAATTCCT
